# Rare Inferior Shoulder Dislocation (Luxatio Erecta)

**DOI:** 10.1155/2015/624310

**Published:** 2015-03-25

**Authors:** Hakan Cift, Salih Soylemez, Murat Demiroglu, Korhan Ozkan, Vahit Emre Ozden, Afsar T. Ozkut

**Affiliations:** ^1^Department of Orthopaedics and Traumatology, Istanbul Medipol University, Istanbul, Turkey; ^2^Department of Orthopaedics and Traumatology, SB. Medeniyet University Goztepe Education and Research Hospital, Istanbul, Turkey; ^3^Acibadem Hospital, Maslak, Istanbul, Turkey

## Abstract

Although shoulder dislocations have been seen very frequently, inferior dislocation of shoulder constitutes only 0.5% of all shoulder dislocations. We share our 4 patients with luxatio erecta and present their last clinical control. 2 male and 2 female Caucasian patients were diagnosed as luxatio erecta. Patients' ages were 78, 62, 65, and 76. All patients' reduction was done by traction-abduction and contour traction maneuver in the operating room. The patients had no symptoms and no limitation of range of motion of their shoulder at their last control. Luxatio erecta is seen rarely, and these patients may have neurovascular injury. These patients should be carefully examined and treated by the orthopaedic and traumatology surgeons.

## 1. Introduction 

Although shoulder dislocations have been seen very frequently, inferior dislocation of shoulder constitutes only 0.5% of all shoulder dislocations [1]. The most inferior dislocations result from forceful hyperabduction of the shoulder. Forceful, direct axial loading of an abducted shoulder can also result in luxatio erecta. The patients come to the emergency room with the hand up position in the effected arm. In this report, we share our 4 patients with luxatio erecta and present their treatment results.

## 2. Case Presentations

### 2.1. Case 1

76-year-old male Caucasian patient presented to the Emergency Department with pain and inability to move his right shoulder. His right arm was locked in abduction of 135 degrees. Because he had dementia we could not get enough information how the injury occurred other than a fall during walking. Examination revealed loss of contour of shoulder, presence of the head of humerus palpable in the axilla. Humeral head was monitored below the glenoid rim in the X-ray. There were no neurovascular deficits and no fracture in the shoulder radiography and humeral head had been seen under the glenoid. Under sedation, reduction was done by traction-abduction and contour traction maneuver. Velpeau bandage was done. Progressive mobilization of shoulder was started after 3 weeks. The patient had no symptoms and no limitation of range of motion of his shoulder at his last control 12 months after reduction.

### 2.2. Case 2

**Figure 1:**
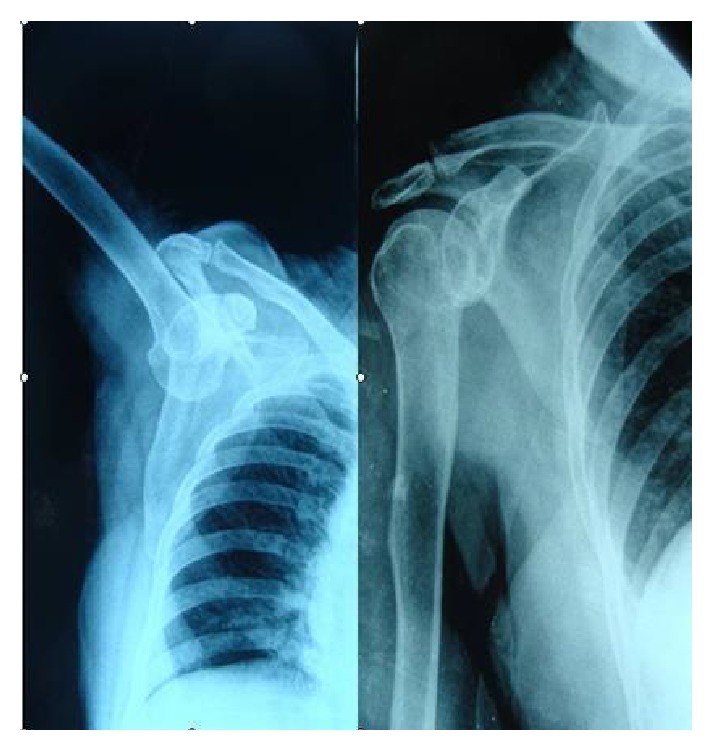
Two consecutive patients, reduction of the previous and next X-rays.

**Figure 2:**
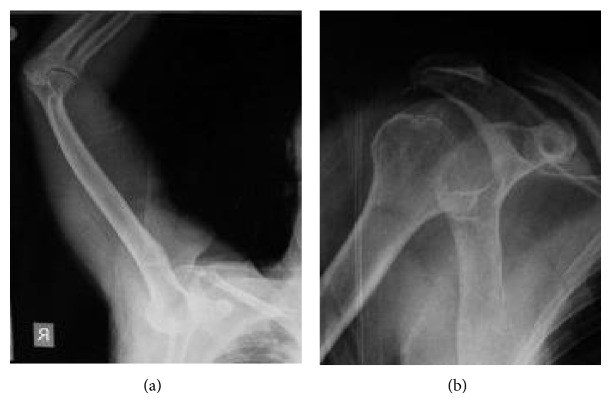
X-ray of the humeral head located below the rim of glenoid and X-ray after the reduction was achieved.

62-year-old female Caucasian patient presented to the Emergency Department with pain and inability to move right shoulder. She was hanged with outstretched hand while she was falling from the wall. She also had Parkinson's disease. She could not move her arm which was elevated and abducted from horizontal plane; prominence of acromion and humeral head was palpable in the axilla. X-ray showed humeral head under glenoid (Figure 1). There were no neurovascular deficits. Under sedation, immediately reduction was done by traction-abduction and contour traction maneuver (Figure 2). Velpeau bandage was applied with 3 weeks of immobilization. The patient had no symptoms and no limitation of range of motion of her shoulder at her last control 8 months after reduction.

### 2.3. Case 3

78-year-old female Caucasian patient presented to the Emergency Department. After car crush, patient fell down the road from the car's hood. Hands this time, it was shoulder level. She had no shoulder motion and humeral head was palpable in the axilla. There was no neurovascular deficit. Under sedation, reduction was achieved with the manipulation of traction of upper limb and counter traction of the trunk. Velpeau bandage was done with 3 weeks of immobilization. The patient had no symptoms and no limitation of range of motion of her shoulder at her last control 14 months after reduction.

### 2.4. Case 4

65-year-old male Caucasian patient presented to the Emergency Department. After car crush, he rolled down from the hood. He had no shoulder motion and humeral head was palpable in the axilla. He had radial paresthesia and reduction was immediately done under sedation. 30 minutes after the reduction radial nerves paresthesia was healed. Velpeau bandage was done with 3 weeks of immobilization. The patient had no symptoms and no limitation of range of motion of his shoulder at his last control 22 months after reduction.

## 3. Discussion

Inferior shoulder dislocation was first described by Middeldorpf and Scharm in 1859 [2].

Several authors described cases of bilateral inferior dislocation [3]. The manuscripts in the English speaking literature have been mostly case series and reports.

Yamamoto et al. presented a total of 102 patients worldwide in their review article [4].

Two mechanisms of injury have been described for luxatio erecta [5, 6]. In the direct mechanism, there is axillary loading on a fully abducted arm and the humeral head is driven through the weak inferior glenohumeral ligaments and joint capsule, frequently fracturing the greater tuberosity and tearing the rotator cuff [6].

In the indirect mechanism, a violent abduction force on an already abducted limb levers the proximal shaft of humerus over the acromion and the humeral head comes to rest below the glenoid in abduction. Neurovascular injuries might also be encountered after luxatio erecta. Although vascular injuries are rare, they are serious and may require surgical intervention with the axillary vessels most commonly involved. Neurological involvement is common, the axillary nerve being the most commonly affected. Neurological injuries recover most of the time within 2 weeks to 1 year [7]. This will probably be done and fast reduction has not been exposed to excessive tension.

Although no fractures occurred in our patients, fracture of the acromion, clavicle, inferior glenoid fossa, and greater tuberosity may occur. In 12% of patients associate tears of rotator cuff could also be seen.

All reductions were achieved under sedation. Surgeon applies axial traction on the abducted humerus at the same time assistant applies counter traction and immobilizes the upper torso. After reduction the dislocated shoulders were immobilized in velpeau bandage for 3 weeks and then pendulum and active assisted exercises were begun. Patients returned to their normal daily activities after 5 weeks with active use of their shoulder.

## 4. Conclusion

Luxatio erecta is seen rarely, and these patients may have neurovascular injury. These patients should be carefully examined and treated by the orthopaedic and traumatology surgeons.

## Abbreviations

Roentgenogram: X-ray.
